# Detection of Nocturnal Elevation in Intraocular Pressure Using a Home Tonometer in a Patient With Iridocorneal Endothelial Syndrome

**DOI:** 10.7759/cureus.42735

**Published:** 2023-07-31

**Authors:** Sayuri Sumi, Sujin Hoshi, Yuta Ueno, Tetsuro Oshika

**Affiliations:** 1 Department of Ophthalmology, Institute of Medicine, University of Tsukuba, Tsukuba, JPN

**Keywords:** aqueous humor drainage, iridocorneal endothelial syndrome, intraocular pressure, home tonometer, glaucoma

## Abstract

We report on a patient with iridocorneal endothelial (ICE) syndrome in whom intraocular pressure (IOP) elevation during the night was detected using a home tonometer. A 44-year-old woman was diagnosed with ICE syndrome in the left eye. Angle-closure attack-like symptoms, including blurred vision and headache, appeared and spontaneously resolved irregularly at bedtime. Daytime examination indicated normal IOP and no obvious signs of glaucoma such as visual field defects or fundus abnormalities. However, nocturnal IOP measurements using a home tonometer revealed temporary high IOP at the time of symptom onset. A home tonometer may be a useful tool to detect transient IOP elevation at night, even if the IOP is normal during daytime examinations.

## Introduction

Iridocorneal endothelial (ICE) syndrome is a spectrum of ophthalmic diseases characterized by abnormalities of corneal endothelial cells, iris, and anterior chamber angles. Epidemiologically, it is more common in young to middle-aged women and is usually uniocular [1]. The pathogenesis involves the production of a Descemet's membrane-like proliferative film that extends and proliferates across the Schwalbe line into the angle and iris surface by abnormal corneal endothelial cells [1]. ICE syndrome is frequently complicated by severe secondary glaucoma, often requiring surgical treatment in relatively young people [2,3]. However, even in patients with undiagnosed glaucoma, temporary elevations of intraocular pressure (IOP) not observed during the day have been reported, and its presence is therefore difficult to confirm in medical clinics [4]. Herein, we report the first case of ICE syndrome in which a hand-held home tonometer (iCare Home®, ICARE FINLAND OY, Vantaa, Finland) was useful in detecting the nocturnal elevation of IOP.

## Case presentation

**Figure 1:**
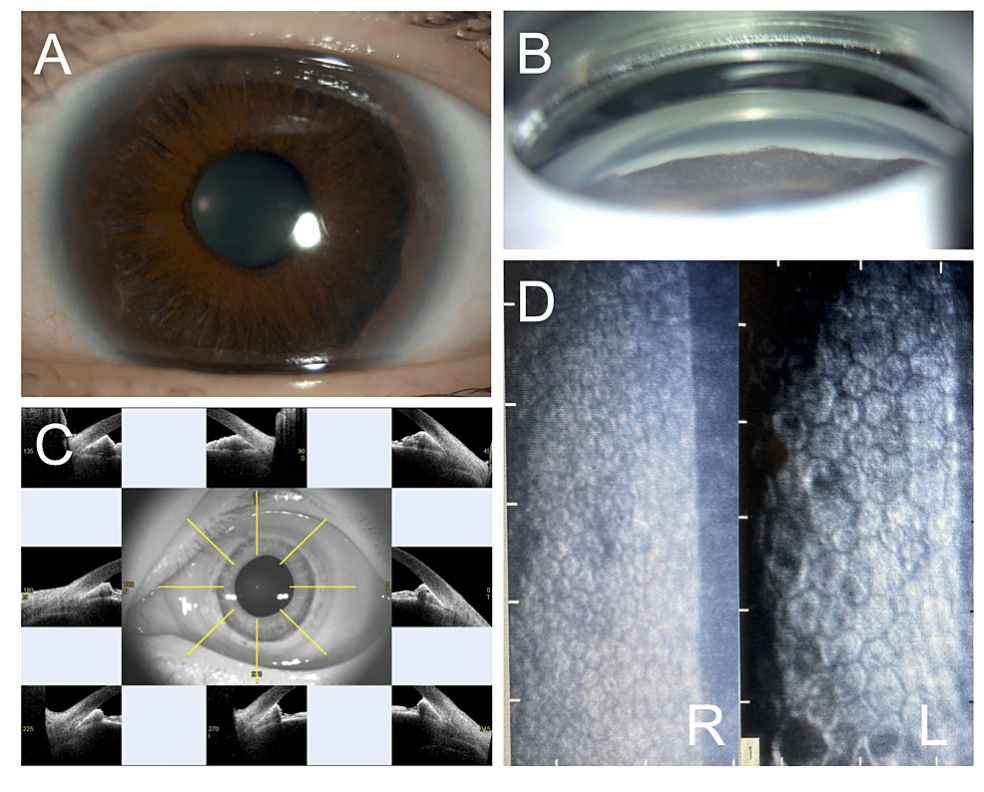
Images of the anterior segment Anterior segment photograph (A), anterior segment-optical coherence tomography (B), and gonioscopy (C) in the left eye showing mild iris atrophy and the full circumference of high peripheral anterior synechiae. Specular microscopy (D) showing normal corneal endothelium in the right eye (R) and decreased cell density, polymorphism, and dark area in the left eye (L).

A 44-year-old woman visited our hospital with mild hyperemia of the left eye. At the initial visit, the best corrected visual acuity was 20/20 in both eyes, with corneal astigmatism of -1.5 D and -2.0 D in the right and left eyes, respectively. The IOP was 18 and 15 mmHg in the right and left eyes, respectively. The left eye had high peripheral anterior synechiae (PAS) covering the entire circumference and mild iris atrophy (Figures 1A-1C). There were no obvious abnormal findings in the optical media and fundus. Corneal endothelial cell density was clearly reduced in the left eye (2,700 cells/mm^2^ in the right eye vs. 1,100 cells/mm^2^ in the left eye) (Figure 1D). Central corneal thickness was 532 and 526 μm in the right and left eyes, respectively. She had no medical or relevant family history. We diagnosed ICE syndrome in the left eye. Optical coherence tomography (OCT) showed no thinning of the peripapillary retinal nerve fiber layer or ganglion cell complex, and assessment using Humphrey automatic visual field meter revealed no visual field defects at the initial and follow-up visits during the observation period.

The follow-up visits in the observation period occurred at 6-month intervals and assessed visual acuity, IOP, and corneal endothelial cell density, during which there were no complaints of vision impairment or symptoms. Approximately seven years later, the patient reported episodes of acute onset headache and blurred vision in the left eye several times a month while falling asleep. These acute angle-closure attack-like symptoms improved within a few hours after resting in a well-lit room. The frequency and duration of symptoms gradually increased. After the symptoms disappeared, visual acuity and IOP measures remained normal during the daytime.

**Figure 2:**
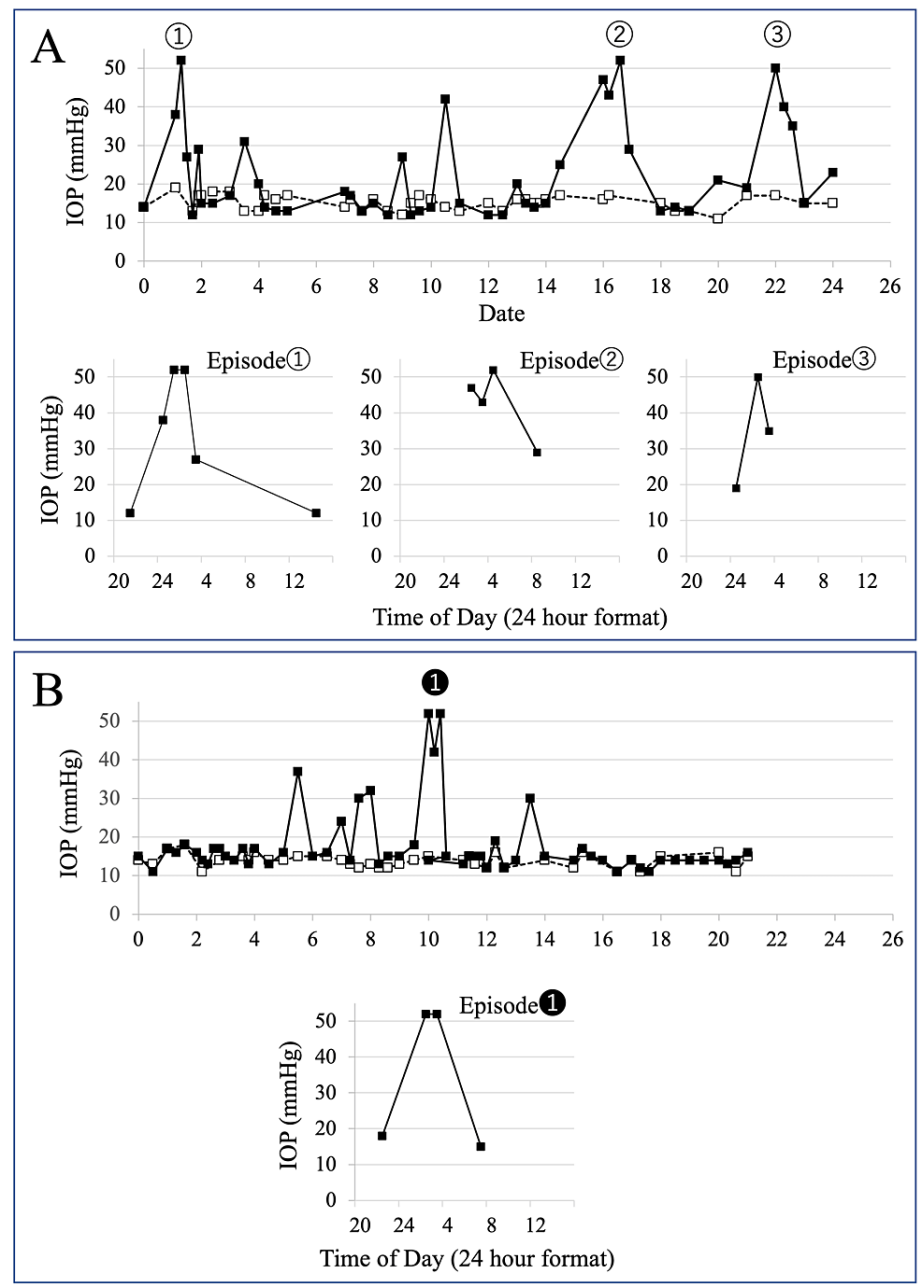
Interocular pressure (IOP) measurements with iCare Home The open squares and dotted line = right eye, solid squares and solid line = left eye. Before treatment (A); after treatment (B). The time courses of the three occasions with the IOP in the left eye > 50 mmHg are shown (lower row). High IOP was associated with headache and vision abnormalities such as blurry vision and inability to focus. These symptoms occurred while falling asleep or immediately after sleeping, lasted two to four hours, and spontaneously improved with rest in a bright room while the IOP decreased. Regarding the frequency of episodes of transient elevations in IOP with acute angle-closure attack-like symptoms, IOP elevations of 20 mmHg or greater occurred in 11 episodes before treatment and decreased to five episodes after the start of treatment, and IOP elevations of 50 mmHg or greater decreased from three episodes before treatment to one episode after the start of treatment.

We suspected transient elevation of IOP as the cause of the short-term headache and blurred vision in the left eye. Thus, for approximately three weeks, the patient was instructed to measure the IOP using iCare Home® at any time during the day or night when she had no symptoms, and frequently when she did. The results are presented in Figure 2A. The right IOP remained generally stable between 10 and 20 mmHg. Although the IOP in the left eye was normal and similar to that of the right eye on most occasions, the left eye had several episodes of high IOP and large IOP fluctuations. The IOP of the left eye was > 50 mmHg during all episodes with symptoms such as blurred vision, eye discomfort, and headache, which occurred three times during the three-week measurement period. When there was no associated blurred vision or headache, discomfort in the eye was perceived at an IOP of approximately 30-40 mmHg. When the presenting symptoms were only headache or vague eye discomfort, the IOP was 25 mmHg or less.

The time courses of the three occasions with the IOP in the left eye > 50 mmHg are shown in Figure 2A (lower row). High IOP was associated with headache and vision abnormalities such as blurry vision and inability to focus. These symptoms occurred while falling asleep or immediately after sleeping, lasted two to four hours, and spontaneously improved with rest in a bright room while the IOP decreased.

Since the symptoms coincided with the occurrence of high IOP, we instructed the patient to take 250 mg of carbonic anhydrase inhibitor (acetazolamide tablet) orally at the onset of symptoms and ripasudil hydrochloride hydrate eye drops (Glanatec ophthalmic solution 0.4%; Kowa Company, Ltd., Tokyo, Japan) twice a day to lower the baseline IOP. After starting the treatment, IOP measurements were taken again using iCare Home® for approximately three weeks (Figure 2B).

A comparison of the IOP in the left eye before and after using ripasudil hydrochloride hydrate eyedrops revealed no significant change in the baseline IOP. However, the frequency of episodes of transient elevations in IOP with acute angle-closure attack-like symptoms decreased (Figure 2B). There was one episode of IOP elevation > 50 mmHg with acute angle-closure attack-like symptoms after starting the treatment (Figure 2B, lower row). Before bedtime at 10:00 p.m., the IOP was 18 mmHg; however, during sleep at 1:00 a.m., the patient developed discomfort and blurred vision in the left eye and headache and was subsequently moved to a bright room. The immediate measurement of IOP in the left eye was > 50 mmHg. The IOP promptly decreased after the oral administration of a 250-mg carbonic anhydrase inhibitor tablet.

Throughout this time, the frequency of acute glaucomatous symptoms had remained unchanged, and the patient had been under consistent monitoring with periodic IOP measurements, ophthalmologic examinations, and visual field tests at our clinic. Written informed consent was obtained from the patient in this study.

## Discussion

It has been reported that 70%-74% of patients with ICE syndrome have glaucoma at the time of initial diagnosis [2,3]. However, even in patients without glaucoma, there may be temporary elevations of IOP that are not observed during the day, similar to the present case and a case reported by Mogil et al. of a patient with ICE syndrome with transient blurry vision associated with transient elevation of IOP on waking that disappeared within one to two hours [4].

Generally, aqueous humor production is higher in the morning than during the rest of the day, and therefore, peak diurnal IOP fluctuation occurs predominantly in the morning hours [5]. Baskaran et al. reported that in patients with primary angle closure and primary angle closure glaucoma, the degree of PAS was associated with diurnal IOP fluctuations [6]. Therefore, Mogil et al. considered that in ICE syndrome, the trabecular meshwork may be obstructed by membranous material formed by abnormal corneal endothelium, which could impair aqueous humor drainage, resulting in high IOP in the morning because of an imbalance in the outflow and production of aqueous humor [4]. In contrast, in the present case, acute angle-closure attack-like symptoms with a sudden increase in IOP occurred at the time of falling asleep or immediately after sleeping, suggesting a different mechanism from that described in the previous report. In this case, despite the presence of a full-length PAS on anterior segment OCT, the baseline IOP was normal, which would be expected to barely preserve the trabecular pathway function. However, the IOP can increase in this case due to structural changes in the corner angle and iris caused by mydriasis in a dark room as well as increased episcleral venous pressure when in a supine position [7-9]. The decrease in symptom frequency after ripasudil hydrochloride hydrate use may also suggest that the drug is effective in patients with ICE syndrome who have preserved primary pathway function. Careful follow-up is needed to determine whether this patient will develop secondary glaucoma in the future as the ICE syndrome progresses.

In this study, we used iCare Home® to measure IOP during symptomatic conditions outside of daytime hours. The home tonometer can be utilized for self-measurement with the understanding that there is limited agreement with the conventional tonometer, especially at lower and higher IOP levels [10,11]. Several studies have reported that IOP fluctuations are an important risk factor for glaucoma progression [9,12-14]. Conventional measurement of diurnal IOP variation requires hospitalization over a 24-hour period and is generally performed approximately every two to four hours; however, it has the following disadvantages: it is burdensome for both the patient and medical personnel, there is a possibility that non-physiological diurnal variation is being measured due to the hospitalization environment, and the reproducibility is not clear. In the present study, the aforementioned disadvantages are mitigated by the use of iCare Home® to measure IOP as needed for three weeks. In addition, it was found that symptoms outside of office hours were attributed to high IOP and that IOP was lowered by moving to a bright room and taking acetazolamide. In this case, the patient measured IOP as needed when symptomatic, although adding regular measurements may also detect high IOP when asymptomatic.

## Conclusions

In patients diagnosed with ICE syndrome, there exist cases where transient intraocular pressure elevations occur repeatedly, even in the absence of distinct glaucoma indicators. Our findings suggest a correlation between symptoms such as transient headaches, nocturnal discomfort, and blurred vision, and a rise in intraocular pressure, as revealed through home tonometry. It is plausible that these recurrent transient IOP elevations, along with their accompanying symptoms, may act as precursors to the development of glaucomatous optic neuropathy in the context of ICE syndrome. Therefore, upon detection of recurrent symptomatic episodes, proactive measures may be required to ascertain if these symptoms are associated with an increase in IOP using a home tonometer.
